# Acute Hydroxychloroquine Overdose With Severe and Prolonged Cardiotoxicity

**DOI:** 10.7759/cureus.88512

**Published:** 2025-07-22

**Authors:** William Richardson, Dan Fisher, Stanley Hassinger, Daniel Culy, Anna Zmuda

**Affiliations:** 1 Emergency Medicine, Prisma Health Midlands, Columbia, USA; 2 Palmetto Poison Center, University of South Carolina College of Pharmacy, Columbia, USA; 3 Emergency Medicine, Trident Medical Center, Charleston, USA; 4 Emergency Medicine, Baptist Health Louisville, Louisville, USA; 5 Emergency Medicine, University of Virginia, Charlottesville, USA

**Keywords:** cardiotoxic agents, covid 19, hydroxychloroquine toxicity, intentional ingestion, medical icu, toxin induced methemoglobinemia

## Abstract

We present a case of an overdose of hydroxychloroquine with severe toxicity and describe the hospital management of the patient. The management of large ingestions can provide significant challenges for the emergency medicine physician, as these patients can present in extremis. A 49-year-old woman presented to the emergency department (ED) two hours following a reported ingestion of 24 grams of hydroxychloroquine in a suicide attempt. The patient developed hypotension, ventricular arrhythmias including torsades de pointes, and profound hypokalemia. She was managed with intravenous fluid resuscitation, mechanical ventilation, vasopressors, electrolyte replacement, high-dose intravenous diazepam, sodium bicarbonate, and lidocaine infusions. The patient was ultimately able to be discharged home neurologically intact. Reported hydroxychloroquine overdoses are relatively infrequent. Understanding the toxicity and management of hydroxychloroquine overdoses is particularly important now, as the prescription rate of hydroxychloroquine increased during the COVID-19 pandemic and has remained high despite the failure of studies to demonstrate a reduction in morbidity and mortality when used for the treatment of COVID-19.

## Introduction

Hydroxychloroquine is used as an anti-inflammatory medication for the treatment of rheumatologic conditions. It was synthesized in 1946 with the addition of a hydroxy group to the parent compound chloroquine in an effort to produce a less toxic compound, yielding a drug with reportedly 40 percent less toxicity than its parent chloroquine [1]. Chloroquine and hydroxychloroquine have demonstrated proarrhythmic effects attributable to inhibition of voltage-dependent sodium (Na^2+^), calcium (Ca^2+^), and potassium (K^+^) ion channels in addition to pacemaker channels, resulting in bradycardia as well as tachycardia, QT interval prolongation, and other conduction blocks [2].

Few cases of severe hydroxychloroquine toxicity are described in the literature [3]. In 2001, Albertson et al. reported only seven cases in the English literature of acute hydroxychloroquine overdose [3]. Severe hydroxychloroquine overdose can cause seizures, coma, hypotension, hypokalemia, and cardiac arrhythmias, including arrest. Traditional treatment of hydroxychloroquine overdose is derived from accepted treatment of chloroquine overdose, including early intubation and mechanical ventilation, activated charcoal, epinephrine, high-dose diazepam infusions, and judicious use of sodium bicarbonate and electrolyte repletion [3,4]. We report a case of a patient surviving a 24 g ingestion of hydroxychloroquine associated with severe and prolonged cardiotoxicity despite aggressive traditional supportive care measures.

## Case presentation

**Figure 1:**
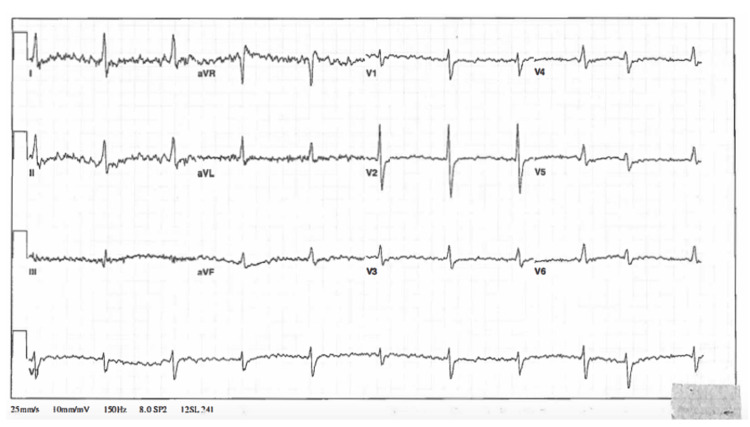
Initial electrocardiogram in the emergency department

A 49-year-old woman with a history of neuropsychiatric systemic lupus erythematosus treated with hydroxychloroquine presented to the emergency department complaining of dizziness, near syncope, and malaise after ingesting hydroxychloroquine. She described intentionally ingesting 120 tablets of 200 mg hydroxychloroquine (24 grams) approximately two hours earlier in a suicide attempt. The patient denied illicit drug use, alcohol consumption, and overdose of other medications. Her other medications included rituximab and prednisone. Initial blood pressure was 55/39 mmHg, pulse 59 bpm, respiratory rate 18 bpm, oxygen saturation 93% on 2 L nasal cannula oxygen, and oral temperature was 36.9°C. Her examination demonstrated a drowsy, tremulous, and ill-appearing woman with a Glasgow Coma Scale of 13 (E3/V4/M6) but was otherwise unremarkable. The patient was initially treated with an intravenous (IV) normal saline bolus, and an electrocardiogram (EKG) was obtained (Figure 1).

Her EKG had tremor-related artifact but did demonstrate a regular rate at 61 bpm, QRS 124 ms, and QTc 610 ms (artifact noted), as well as nonspecific ST-T wave changes. Despite two liters of 0.9% normal saline infusion, the patient’s blood pressure worsened, and she became obtunded, requiring intubation for airway protection. After intubation, a 20 mcg/min continuous infusion of norepinephrine was initiated along with additional 0.9 percent normal saline boluses (4 L of 0.9 percent normal saline in total), resulting in a transient improvement in her blood pressure to 132/93 mmHg. In addition, IV diazepam 160 mg (approximately 2 mg/kg) was infused over six hours, epinephrine infusion (20 mcg/min) was started for vasopressor support, and a sodium bicarbonate continuous infusion was initiated, given evidence of QRS prolongation. Given the time of ingestion was an approximation, and given the possibility of co-ingestants with delayed absorption, activated charcoal was administered via orogastric tube following intubation.

**Figure 2:**
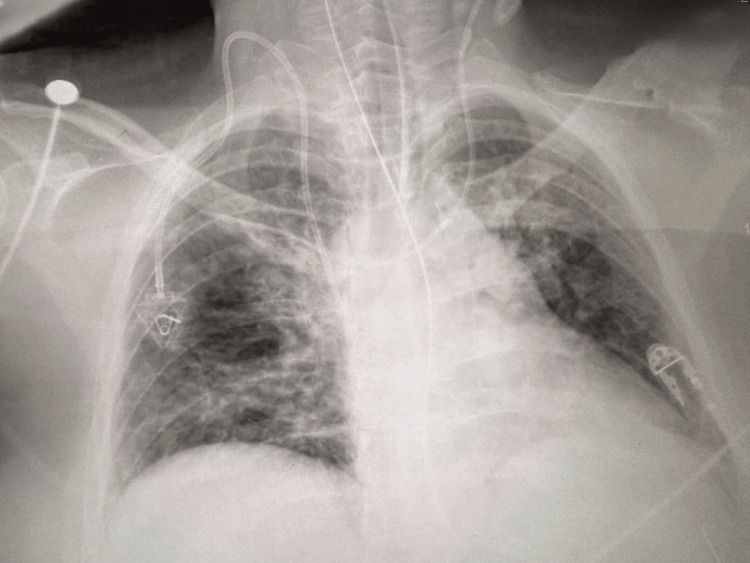
Patient's post-intubation chest X-ray showing mild pulmonary edema

Emergency department laboratory studies were pertinent for a serum potassium concentration of 2.8 mEq/L, lactate 3.37 mmol/L, and serum creatinine 1.5 mg/dL (unknown baseline). Other electrolyte values, hemoglobin, hematocrit, and troponin were initially unremarkable. Additionally, ethanol, salicylate, and acetaminophen concentrations were undetectable (Table 1). A urine drug screen was also unremarkable and did not suggest other drug exposures (Table 2). To address the patient’s hypokalemia, two IV doses of 10 mEq KCl were administered via central line over one hour. The patient’s chest X-ray obtained after intubation in the ED showed mild pulmonary edema (Figure 2).

**Table 1:** Initial laboratory results

Lab (serum level)	Patient's value	Reference range
Sodium	145 mEq/L	136-145 mEq/L
Potassium	2.8 mEq/L	3.6-5.1 mEq/L
Chloride	108 mEq/L	101-111 mEq/L
HCO_3_ (bicarbonate)	22 mEq/L	22-32 mEq/L
Blood urea nitrogen	24 mg/dL	6-20 mg/dL
Creatinine	1.5 mg/dL	0.4-1.2 mg/dL
Glucose	64 mg/dL	70-100 mg/dL
Ionized calcium	1.27 mmol/L	1.16-1.32 mmol/L
Anion gap	13.0	3.0-13.0
Troponin	0.00 ng/mL	0.00-0.08 ng/mL
Hemoglobin	15.6 g/dL	11.5-14.5 g/dL
Hematocrit	46.0%	34.0-43.0%
Lactate	3.37 mmol/L	0.5-2.0 mmol/L
Ethanol	undetectable	< 10 mg/dL
Salicylate	< 4.0 mg/dL	< 30 mg/dL
Acetaminophen	< 10.0 mcg/dL	10.0-30.0 mcg/dL

**Table 2:** Urine drug screen

Lab	Patient's value	Reference range
Opiates	None Detected	None Detected
Barbiturates, qualitative	None Detected	None Detected
Phencyclidine (PCP)	None Detected	None Detected
Amphetamine	None Detected	None Detected
Cocaine screen	None Detected	None Detected

**Figure 3:**
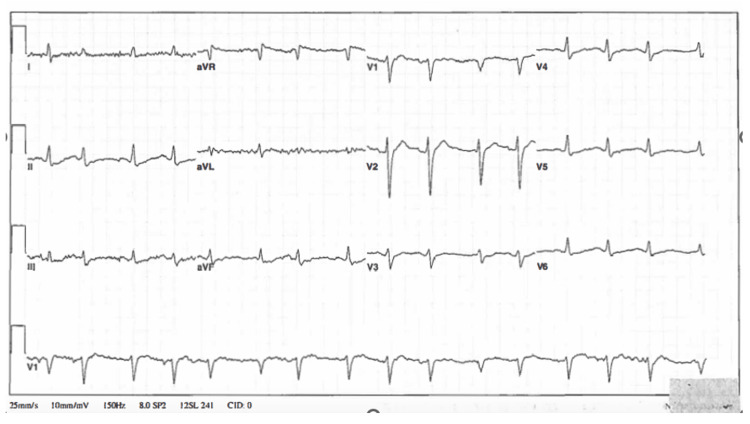
Electrocardiogram in the ICU obtained approximately 10 hours after initial ED presentation showing a QRS of 120 ms

In the intensive care unit (ICU), the patient required increasing and varying doses of norepinephrine and epinephrine infusions to maintain a mean arterial pressure greater than 65 mmHg. IV sodium bicarbonate and diazepam infusions were continued. During the first 24 hours of admission, the patient developed multiple episodes of pulseless monomorphic ventricular tachycardia and torsades de pointes but did achieve return of spontaneous circulation after emergent defibrillation and 2 g IV magnesium sulfate. A repeat EKG (Figure 3) obtained in the ICU showed an estimated QTc of 495 and QRS of 120 ms. She developed a fever of 102.6°F, and broad-spectrum antibiotics were administered along with hydrocortisone 100 mg IV q8 hours due to her history of chronic prednisone use. 

Early during the patient’s ICU stay, she was on five titrated medications simultaneously for blood pressure support: epinephrine 20 mcg/min, norepinephrine 20 mcg/min, vasopressin 0.04 units/min, phenylephrine 240 mcg/min, and dopamine 400 mcg/min. An infusion of lidocaine was also initiated. Her peak documented QT was 708 ms, which ultimately normalized with electrolyte repletion and time. QRS duration normalized to 90 ms by hospital day two on the sodium bicarbonate infusion. Early in her admission, the patient developed acute kidney injury with a peak serum creatinine of 3.4 mg/dL, oliguria, hypokalemia with a nadir of 1.1 mEq/L despite potassium repletion on hospital day one, and hypomagnesemia of 1.4 mg/dL (Table 3). She was briefly weaned from the ventilator on hospital day six but required reintubation that same day due to hypoxia and respiratory distress secondary to persistent pulmonary edema. On hospital day seven, she developed methemoglobinemia, which was suspected to have been secondary to the IV lidocaine infusion. Methemoglobin concentration peaked at 18 percent, lidocaine was discontinued, one percent methylene blue therapy was initiated with a 90 mg IV bolus (approximately 1 mg/kg), and her methemoglobinemia rapidly resolved. A serum hydroxychloroquine concentration collected from a specimen on hospital day four returned elevated at 1006.3 ng/mL (Table 3). For patients with systemic lupus erythematosus, the proposed target concentration is 1000 ng/mL. No serum concentration was obtained during the initial presentation.

**Table 3:** Laboratory results during the first week of hospitalization

Lab (serum level)	Patient's value	Reference range
Creatinine, peak	3.4 mg/dL	0.4-1.2 mg/dL
Potassium, nadir	1.1 mEq/L	3.6-5.1 mEq/L
Magnesium, nadir	1.4 mg/dL	1.6-2.6 mg/dL
Methemoglobin	18 percent	0-2 percent
Hydroxychloroquine, day 4	1006.3 ng/mL	Target: 1000 ng/mL

During the patient’s hospitalization, her creatinine trended back to its admission baseline, urine output normalized, and she did not require renal replacement therapy. Her cardiopulmonary status improved without further cardiac arrhythmias, and she was successfully weaned from the ventilator. The patient was deemed medically stable, and following inpatient psychiatric evaluation, was discharged on hospital day 17 to follow up with her primary care provider and psychiatrist.

## Discussion

Reports of hydroxychloroquine overdose and severe toxicity are rare despite its small toxic-to-therapeutic margin. However, its use rose dramatically with its controversial and unsubstantiated application as a therapeutic agent during the COVID-19 pandemic [5,6]. In the week of March 15-21st, 2020, the prescription fill rates for 28 tablets of hydroxychloroquine increased by nearly 2000 percent compared to the same week in 2019 [5,6]. This placed hydroxychloroquine in many more homes than it otherwise would have been. Hydroxychloroquine has been shown to have a time to peak effect of two to four and a half hours and a terminal elimination half-life of up to 40 days [7]. Rapid deterioration following overdose can occur from cardiotoxic effects, including hypotension, conduction abnormalities from sodium, potassium, and calcium channel blockade, and neurologic toxicity [8]. The threshold for toxicity or for lethal ingestion is unknown, but ingestions over 2-4 g have been associated with severe toxicity [9,10]. Fatalities have been reported following ingestions of approximately 12 g, a 29-year-old woman ingesting 14 g, and a two-year-old ingesting 12 g [3,11].

This case highlights several treatment modalities described in the literature for successfully treating severe hydroxychloroquine overdose. Due to the similarities between chloroquine and hydroxychloroquine overdoses, most treatment regimens are based on reported experiences with chloroquine toxicity. The most severe effects include profound hypotension, hypokalemia, QRS widening, QTc prolongation, cardiac arrhythmias, seizures, and coma. As noted in previous studies regarding chloroquine, high-dose diazepam has been suggested to be cardioprotective as well as providing anticonvulsant activity and antiarrhythmic support [12]. Initial bolus dosing of diazepam up to 1-2 mg/kg followed by infusion of 1-2 mg/kg/day has been recommended, though there is variability in the literature, with infusions of up to 6 mg/hr reported as well [13,14]. Furthermore, there is literature supporting combining high-dose diazepam with early and aggressive epinephrine infusions, activated charcoal, and early intubation with mechanical ventilation [3,4]. In this case, the patient presented profoundly hypotensive, and despite fluid resuscitation, the patient decompensated further, requiring intubation and ultimately multiple vasopressors. Some experience also suggests that epinephrine is the vasopressor of choice in chloroquine or hydroxychloroquine poisoning, but this patient required as many as five vasopressors for pressure support throughout her hospital course. In the emergency department, the patient received 160 mg of diazepam infused over six hours, then a second infusion of 160 mg of diazepam over the subsequent 24 hours. No seizures were seen in this case. A 1006.3 ng/mL serum hydroxychloroquine concentration obtained during hospitalization confirmed ingestion, although this was four days after ingestion.

Another well-documented complication of hydroxychloroquine poisoning is hypokalemia. Uncertainty exists regarding the optimal endpoint of potassium repletion, as the hypokalemia seems to be secondary to intracellular shifting rather than total body depletion. Profound hypokalemia can cause cardiac arrhythmias. Thus, careful repletion to normal serum concentrations is a reasonable goal while carefully monitoring potassium levels as the resuscitation progresses, since rebound hyperkalemia has been reported [15]. In this case, the patient initially had a potassium concentration of 2.8 mEq/L, but this decreased to 1.1 mEq/L before improvement was seen. Potassium supplementation occurred as needed with a goal to keep concentrations within the normal range. Although sodium bicarbonate infusion can potentially worsen hypokalemia, the risk of arrhythmia suggested by the widened QRS interval from hydroxychloroquine-associated sodium channel blockade and worsening acidosis was thought to outweigh this risk. Hypokalemia has been reported to occur early following hydroxychloroquine overdoses and to correlate with the severity of toxicity [3].

Despite early aggressive therapy, the patient did have several episodes of ventricular tachycardia, including torsades de pointes, on hospital day one. A striking aspect of this case was the ongoing cardiac dysrhythmias during the first several days of hospitalization. Typically, cardiotoxic effects occur early but do not last longer than 24 hours [3]. It is reasonable to suspect that these ongoing dysrhythmias were the result of the notably large hydroxychloroquine ingestion in this particular case. Additionally, the repeated episodes of cardiac arrest requiring advanced cardiac life support (ACLS) may have contributed to an ongoing state of cardiac sensitivity or irritability.

On hospital day seven, the patient developed methemoglobinemia. This was presumably a result of the lidocaine infusion over several days of ICU care. The methemoglobinemia completely resolved following administration of methylene blue and cessation of the lidocaine infusion. It should be noted that chloroquine and other quinine derivatives are potential precipitants of methemoglobinemia [7,16]. With the rise in hydroxychloroquine use during the early part of the COVID-19 pandemic, Sahu et al. noted that among eight known cases of COVID-19-associated methemoglobinemia, seven had received hydroxychloroquine [17]. Due to its structural similarity to chloroquine, and given the associations noted during the COVID-19 pandemic, it is plausible to suggest that hydroxychloroquine may have contributed to the development of methemoglobinemia in this case.

A serum hydroxychloroquine concentration of 1006.3 ng/ml was obtained on hospital day four. The half-life of hydroxychloroquine in overdose is reported to range from 15.5 to 31 hours [18]. Thus, several half-lives passed before a blood sample was collected to test the patient’s serum concentration of hydroxychloroquine. This suggests a much higher serum concentration existed at the time of the patient’s arrival at the hospital and correlates with the severe clinical course the patient experienced. 

Because of the high volume of distribution (Vd is 78 L/kg) and significant protein binding, hemodialysis is not likely to be an effective treatment in hydroxychloroquine overdose [7]. Activated charcoal has been shown in a volunteer study to absorb 95-99% of ingested chloroquine when administered early. Thus, activated charcoal should be strongly considered if airway protection is established [19]. Lipid emulsion therapy may be considered as well. It has been used in limited cases of hydroxychloroquine overdose, with the majority of patients who received it surviving to hospital discharge [20]. However, at this time, there is insufficient evidence to positively recommend its use in hydroxychloroquine poisoning.

## Conclusions

Because large hydroxychloroquine overdoses are rarely reported, this case adds another experience to the presentation, complications, and management following a massive hydroxychloroquine ingestion. This case is especially interesting as it is an isolated hydroxychloroquine overdose without co-ingestions. 

Management includes early treatment of hypotension with IV fluids and vasopressors, airway protection, management of cardiac dysrhythmias, and cautious potassium repletion. The patient in this case survived to hospital discharge after a reported 24 g ingestion of hydroxychloroquine, which would be expected to have a high mortality rate. Aggressive supportive care, to include early intubation with mechanical ventilation, epinephrine and other vasopressor infusions, and high-dose diazepam dosing, was used successfully in this case. In a single case, it is impossible to state which of these life-saving treatments provided the most benefit; however, the combination of aggressive care measures resulted in survival and discharge from the hospital. 
